# Studies on Interaction of Buffalo Brain Cystatin with Donepezil: An Alzheimer's Drug

**DOI:** 10.1155/2013/842689

**Published:** 2013-08-25

**Authors:** Fakhra Amin, Bilqees Bano

**Affiliations:** Department of Biochemistry, Faculty of Life Sciences, Aligarh Muslim University, Aligarh 202002, Utar Pradesh, India

## Abstract

When drugs bind to a protein, the intramolecular structures can be altered, resulting in conformational change of the protein. Donepezil, an Acetyl Cholinesterase inhibitor (AChE), is commonly prescribed to patients with Alzheimer's disease (AD) to enhance cholinergic neurotransmission. It is the “first-line” agents in the treatment of Alzheimer's disease used to improve cognitive function in the disease. In the present study, a cysteine protease inhibitor (cystatin) has been isolated from buffalo brain using alkaline treatment, 40 to 60% ammonium sulphate fractionation and gel filtration chromatography on Sephadex G-75 with % yield of 64.13 and fold purification of 384.7. The purified inhibitor (Buffalo Brain Cystatin, (BBC)) was eluted as a single papain inhibitory peak which migrated as single band on native PAGE; however, on SDS-PAGE with and without beta mercaptoethanol (**β**ME) BBC gave two bands of M W 31.6 and 12.4 KDa, respectively. The molecular weight determined by gel filtration came out to be 43.6 KDa. The UV spectra of cystatin on interaction with donepezil suggested a conformational change in the protein. The fluorescence spectra of BC-donepezil composite show structural changes indicating 40 nm red shift with significant increase in fluorescence intensity of cystatin in the presence of donepezil representing an unfolding of cystatin on interaction, which is an indication of side effect of donepezil during the use of this drug.

## 1. Introduction

 Brain is exposed to a variety of neuromodulating agents given as therapy. The effect of these agents apart from generation of desired activity must be assessed to understand the mechanism of action and side effects of the drug if any.

Alzheimer is the most common cause of dementia; it is a primary degenerative disease of the brain. Onset is usually late in life with increasing impairment of memory, cognition, linguistic ability, and judgment. It is a progressive brain disorder that gradually destroys a person's ability to learn, reason, and carry out daily activities [1, 2].

**Figure 1:**
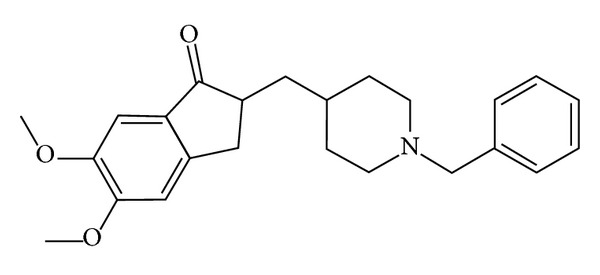
Structure of donepezil [17].

 Acetylcholine is an important neurotransmitter associated with normal functioning of the brain. The greatly reduced concentration of acetylcholine in the cerebral cortex is a significant factor in AD [3, 4]. People with Alzheimer disease (AD) have lower level of acetylcholine with the development of abnormalities in cholinergic neurons. One approach to lessening the impact of these abnormalities is to inhibit the breakdown of acetylcholine (Ach) by blocking the relevant enzyme AchE (acetyl choline esterase) [2] (Figure 1). 

ChE inhibitors prevent the hydrolysis of ACh to choline and acetate in the synaptic clefts and result in activating cholinergic transmission [3].

Donepezil is a piperidine-class AChE inhibitor, rationally designed especially for AD [5, 6]. Donepezil was proven to improve cognitive function of mild to severe moderate AD patients and showed excellent tolerability without hepatotoxicity [7–9]. 

Donepezil is the first agent to be successfully developed specifically for the treatment of cognitive decline associated with Alzheimer's disease; it is marketed under the trade name of Aricept and works as an acetyl cholinesterase inhibitor [10]. It has an oral bioavailability of 100% and easily crosses the blood-brain barrier. It has a half-life of about 70 hours. It is believed that donepezil works by reducing the breakdown of acetylcholine thus increasing the concentration of acetylcholine in the brain reverting it back to its normal function [11]. 

 Recent researches have shown that cathepsins are involved the processing of certain neuropeptides in the central nervous system (CNS) [12] where cystatin C is also present in high concentration and their concentration is suggested to play an important role in brain diseases. An imbalance between the activity of cathepsins and cystatins may lead to accumulation of potentially amyloidogenic fragments which aggregates and forms amyloid fibrils in nerve cells of AD brain in which cystatin concentration decreases. Potentially amyloidogenic fragments generated by imbalance of cathepsins and cystatin are released into the extracellular space. In normal persons, cysteine proteinases help in *β*-peptides clearing [12]. 

 Cystatins constitute a powerful regulatory system for endogenous cysteine proteinases (cathepsins) which are often secreted or leaking from the lysosomes of dying or diseased cells [13]. They are the natural inhibitors of cysteine proteases which belongs to a super family of proteins with wide occurrence in tissues and cells [14]. On the basis of homology, inhibition of target enzymes, and presence or absence of disulphide bonds, cystatin super family has been divided into three families.

 Family 1: also called as stefins include members of low molecular weight proteins (11 kDa) which lack disulphide bonds and carbohydrate contents. This family includes cystatin A, B, stefins C, and stefins D.

 Family 2: known as cystatin family represented by the inhibitors of a bit larger molecular weight proteins (13 kDa) as compared to stefins and possesses disulphide bond towards the carboxyl terminal with no carbohydrates. They are found both in the cells and body fluids. Common example is cystatin C.

 Kininogens or family 3 cystatins are large precursor molecules of the vasoactive kinins. They are single chain glycoproteins, which serve a variety of biological functions such as kinin delivery, induction of endogenous blood coagulation cascade, and mediation of the acute phase response. They are found only in blood plasma [15].

Cystatins tightly bind and inhibit the activity of cathepsins, if the activity of cathepsins is not regulated it will lead to chronic diseases [13]. It has been probed that proteinases and their endogenous inhibitors cystatins are closely associated with senile plaque, cerebrovascular amyloid deposits, and neurofibrillary tangles in Alzheimer disease [1].

**Figure 2:**
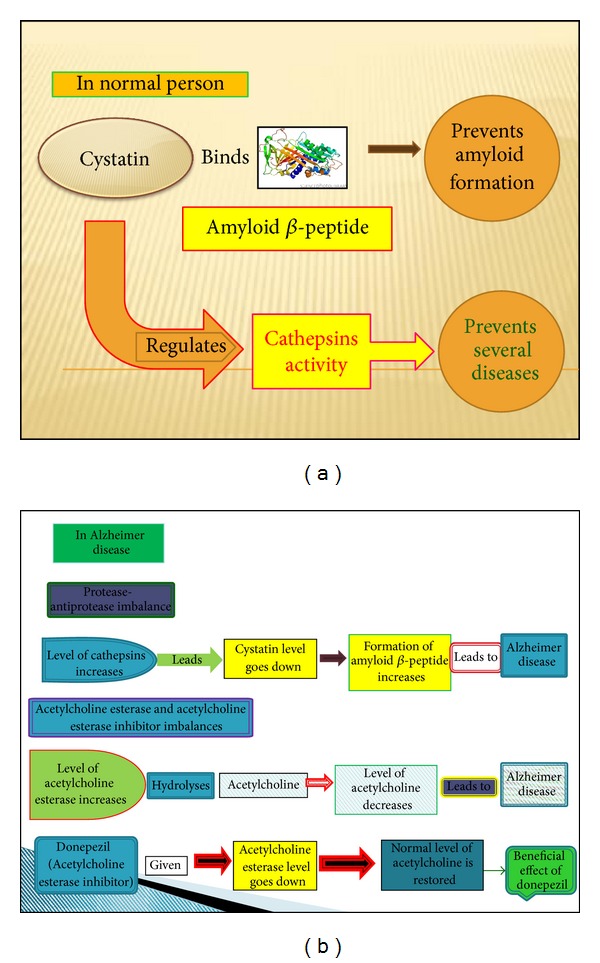
Proposed work.

In normal person, cystatins bind to cathepsins and prevent amyloid formation; however in AD, the level of cystatin goes down and the level of cathepsins and AChE enzyme increase which leads to decreases in acetylcholine and leads to formation A*β* peptides, donepezil when supplemented it binds to AChE and prevents acetylcholine breakdown thus increases the level of Acetyl choline which is required for normal functioning of the brain [16]. An earlier report showed that donepezil binds with HSA and changes its free concentration in plasma showing the possibility of conformational change in protein (Figure 2) [17]. 

 The aim was to find out if supplementation of donepezil has any effect on the activity of cystatin (major regulator of thiol proteases cathepsins B, H, and L, etc.) in the mammalian system. If the activity of these proteases is not regulated, it will lead to protease and antiprotease imbalance, a cause of several diseases [18].

So we have investigated whether cystatin binding with donepezil has any role in the proper action of drug or leads to what kind of side effects as well as to gain knowledge about any conformational change in cystatin effecting its activity.

Our study shows that donepezil unfolds cystatin which may not be able to bind to cathepsins; therefore an imbalance of protease - antiprotease occurs in the presence of donepezil leading to considerable side effect of the drug as cystatins play significant role in several diseases like arthritis cancer and cardiovascular diseases [19]. Therefore the usage of donepezil in such patients requires further attention.

## 2. Material and Methods

### 2.1. Experimental Procedures

#### 2.1.1. Materials

Papain (99% purity) was obtained from Sigma Chemical Company (St. Louis, USA). Donepezil (an Alzheimer drug) was purchased from Ranbaxy (India). The solutions were prepared in 50 mM phosphate buffer of pH 7.4. Salts were purchased from Merck (India). The protein concentration was determined spectrophotometrically. All other reagents were of analytical grade, and double distilled water was used throughout.

#### 2.1.2. Methods

*Purification of Brain Cystatins*. Buffalo brain whole mass (150 g) was brought fresh from slaughter house in an ice bucket. It was thoroughly washed with water, thin membrane and nerves were removed by forcep, and the whole brain tissue was homogenized in 50 mM sodium phosphate buffer (300 mL) of pH 7.5 containing 0.15 M NaCl, 3 mM EDTA, and 2% n-butanol. After centrifugation at 11000 rpm for 15 minutes at 40°C, residue was discarded and the supernatant was further processed. The procedure involved a combination of alkaline treatment (pH 11.0), ammonium sulphate fractionation, and gel filtration chromatography. Buffalo brain was homogenized and fractionated with ammonium sulfate between 40 and 60%; it was then dialyzed against 50 mM sodium phosphate buffer pH 7.4 containing 0.1 M NaCl. Elution profile showed two protein peaks one major and one minor called as peak-I and peak-II. Peak-I corresponding to high molecular weight Buffalo Brain Cystatin had significant inhibitory activity and protein content; however, peak-II with insignificant proteins concentration and low inhibitory activity was not taken into consideration for further studies. Peak-I named as BC was purified with fold purification of 384.72 and yield of 64.13%. Papain inhibitory fractions of peak-I were pooled, concentrated, and checked for purity. Five milliliter fractions were collected and assayed for protein and cystatin activity. Homogeneity of the preparation was investigated by 7.5% PAGE [20].

### 2.2. Interaction of Donepezil with the Cystatin

#### 2.2.1. Spectroscopic Studies

*Fluorescence Spectra of Brain Cystatin with Drug*. Brain cystatin (BC) (1 *μ*M) was incubated for 30 min with increasing concentrations of donepezil in 0.05 M sodium phosphate buffer pH 7.5 in a final reaction volume of 1 mL at room temperature. Drug solutions were prepared in the same buffer. Fluorescence measurements were carried out on a Shimadzu Spectrofluorometer model RF-5301PC (Shimadzu, Japan) equipped with a 150 W Xenon lamp and a slit width of 10 nm at 298 K. The fluorescence was recorded in wavelength region 300–400 nm after exciting the protein at 280 nm. The slits were set at 10 nm for excitation and emission. The path length of the sample was 1 cm.

*UV Spectra of Cystatin in the Presence of Donepezil*. The UV measurement of brain cystatin in the presence and absence of drug was made in the range of 200–300 nm, and the inhibitor (cystatin) concentration was fixed at 1 *μ*M while the drug concentration was varied from 0.16 *μ*M−1.6 *μ*M. Absorption spectra were recorded on a double beam Shimadzu UV-vis spectrophotometer UV-1700 using a cuvette of 1 cm path length.

*Activity Measurement of Brain Cystatin in the Presence of Donepezil*. The inhibitory activity of the purified inhibitor (BC) under native conditions was assessed by its ability to inhibit caseinolytic activity of papain by the method of Kunitz [21]. The inhibitor (1 *μ*M) was incubated with increasing concentrations of donepezil at 25°C for 30 min before the activity was measured. Activity of untreated BC was taken as 100%.

## 3. Results

### 3.1. Interaction of Donepezil with Brain Cystatin

 Alzheimer disease is a progressive brain disorder that gradually destroys a person's memory and ability to learn, reason, and make judgments. AChE is responsible for degradation of the neurotransmitter acetylcholine (ACh) in the synaptic cleft of neuromuscular junctions and of neuronal contacts in the central nervous system [22, 23]. Donepezil belongs to the important class of acetyl cholinesterase inhibitors (AChEIs) [24]. The results of the interaction of donepezil with cystatin are given below.

### 3.2. Intrinsic Fluorescence Studies of Cystatin in the Presence of Donepezil

**Figure 3:**
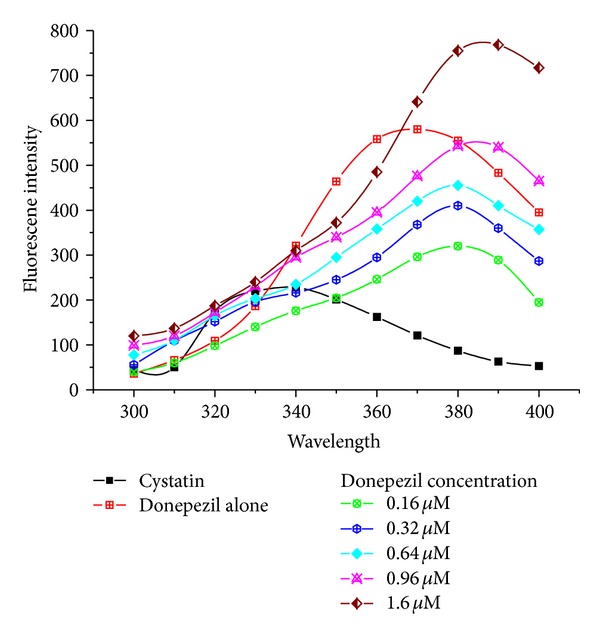
Fluorescence spectra of cystatin in the presence and absence of donepezil.

 Cystatin (1 *μ*M) was incubated with various concentrations of donepezil varying from 2 to 10 *μ*M for 30 min. The fluorescence was recorded in the wavelength region of 300–400 nm after exciting the protein solution at 280 nm for total protein fluorescence. Donepezil caused unfolding of the cystatin as indicated by enhancement in fluorescence intensity accompanied by the red shift of 40 nm as compared to *λ*_max⁡_ of native cystatin (340 nm) while the drug (native) shows *λ*_max⁡_ at 370 nm. However at 1.6 *μ*M, when it forms complex with cystatin there was shift in *λ*_max⁡_ of 10 nm with significant enhancement in fluorescence intensity (Figure 3).

Cystatin (1 *μ*M) was incubated with various concentrations of donepezil varying from 0.16 *μ*M to 1.6 *μ*M for 30 min. The fluorescence was recorded in the wavelength region 300–400 nm after exciting the protein solution at 280 nm for total protein fluorescence. The slits were set at 10 nm for excitation and emission. The path length of the sample was 1 cm in the final reaction volume of 1 mL in 0.05 M sodium phosphate buffer pH 7.5. 

### 3.3. UV-vis Spectra of Cystatin in the Presence and Absence of Donepezil

 Cystatin concentrations were fixed at 1 *μ*M while the donepezil concentrations varied from 0.16 *μ*M to 1.6 *μ*M. Absorption spectra of native cystatin and in the presence and absence of donepezil were recorded in the range of 200–300 nm. The UV absorption intensity of cystatin increased with increasing concentration of donepezil concentration; however the slight decrease in absorption intensity may be due to disruption or perturbation of absorbing groups (Figure 3).

Cystatin concentrations were fixed at 1 *μ*M while the donepezil concentration was varied from 0.16 *μ*M to 1.6 *μ*M. Absorption spectra of native cystatin in the presence and absence of donepezil were recorded in the range of 200–300 nm in a cuvette of 1 cm path length for 30 min in the final reaction volume of 1 mL in 0.05 M sodium phosphate buffer pH 7.5.

### 3.4. Inhibitory Activity of Cystatin in the Presence of Donepezil

 A change in the inhibitory activity of cystatin with increasing concentration of donepezil is shown in (Table 1). Effect of Donepezil on cystatin function was assessed by monitoring its changes in antiproteolytic activity by caseinolytic assay of papain [21]; 1 *μ*M of cystatin was incubated with increasing concentration of donepezil (0.16–1.6 *μ*M). Exposure of cystatin to increasing concentration of donepezil resulted in rapid decline of antiproteolytic activity; 85% decline in the activity was seen at 1.6 *μ*M of donepezil with more than half of the inactivation of cystatin was taking place at concentration as low as 0.32 *μ*M.

Table 1 shows changes in the inhibitory activity of brain cystatin after its incubation for its inhibitory activity with increasing concentrations of donepezil. Cystatin (1 *μ*M) was treated with varying concentration of donepezil (0.16 *μ*M–1.6 *μ*M) for 30 min in the final reaction volume of 1 mL in 0.05 M sodium phosphate buffer pH 7.5. 

All data are expressed as mean ± S.E. for three different sets of experiments; statistical significance was conducted employing ONE WAY ANOVA. A probability level of 0.05 was selected showing that results are significant.

## 4. Discussion

Drug inducing changes in protein function leading to adverse side effects is the area of continual scientific investigation [25, 26]. 

Even small structural differences in protein conformation can lead to drastic changes in functional parameters [26]. Addition of small molecules such as many drugs, particularly those with local anesthetics, tranquillizers, and antidepressants, can bind to the native state and can alter the delicate balance of various interactions in proteins [27–30].

Accumulation of drug molecules at certain sites in the body causes their localized high concentration leading to adverse drug reactions [31] and ligand induced protein structure conformational changes [32]. These are major problems complicating drug medical therapy; therefore investigations which combine the study of the conformational changes in proteins through variations of external parameters between proteins and drugs and the structure of the resulting complexes are of particular interest. These studies enable to elucidate how ligand affinity is regulated and how the protein conformation is altered upon complexation [26] which is of crucial importance in a vast range of important biochemical phenomena.

In the present work, structural and functional analysis of cystatin, a protein ubiquitously present in mammalian cells and tissues, was studied which showed a significant increase in fluorescence intensity due to unfolding of cystatin in the presence of donepezil (Figure 3). Such kind of changes have also been documented earlier, after interaction of ligands (phytohormones, cytokinins, abscisic, and gibberellic acids) with wheat germ agglutinin resulting in 60% increase in fluorescence intensity of native protein [33].

**Figure 4:**
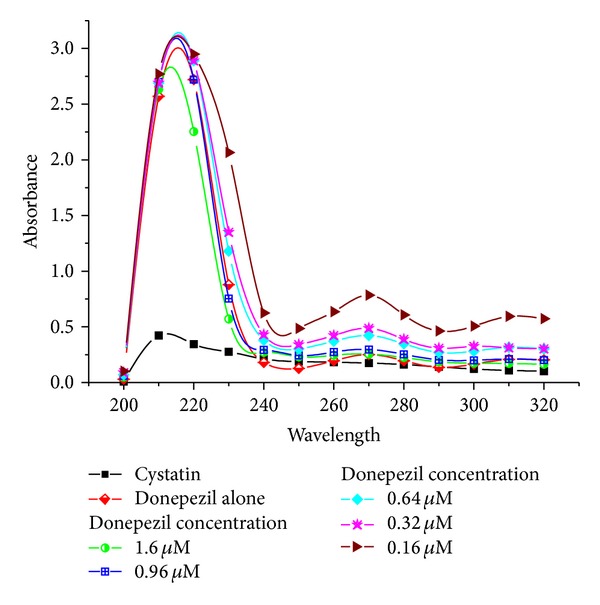
UV-vis spectra of cystatin in the presence and absence of donepezil.

Donepezil-cystatin complexation showed 40 nm red shift in *λ*_max⁡_ indicating exposure of aromatic residues to the solvent caused by conformational changes in the protein [34, 35]. Absorption spectral measurements of cystatin in the presence of drug showed a peak noticeable at 275 nm and 210 nm in spectra obtained at 0.16 *μ*M donepezil (Figure 4). suggesting changes mainly due to tryptophan and tyrosine residues [36]. There was a gradual decline in cystatin activity with increasing drug concentration resulting in 62% loss at 0.64 *μ*M donepezil (Table 1). Further magnitude of decline was relatively smaller with increasing drug concentration at 1.6 *μ*M; cystatin the inhibitor retained only 15% of its antiproteolytic potential. 

 Thus, the results indicate that the UV absorption and fluorescence emission changes in donepezil mediated interaction are due to conformational changes in cystatin mainly arising from interaction affecting the chromophoric groups of the protein which produce significant effect on the activity of cystatin. The study shows that in the presence of donepezil, cystatin gets unfolded which is a side effect of donepezil. 

The knowledge about the pharmacokinetics and pharmacodynamics of the drug-protein interaction continues to expand. The increased information available to clinicians might help in optimizing the use of these agents in the management of patients with Alzheimer's and other diseases. The clinical utility of measuring these parameters in daily practice awaits further research [37].

## Figures and Tables

**Table 1:** Inhibitory activity of cystatin in the presence of donepezil.

S. no	Drug concentration	% Remaining inhibitory activity of cystatin
1	Cystatin alone	100
2	Cystatin + 0.16 *µ*M donepezil	57 ± 0.623
3	Cystatin + 0.32 *µ*M donepezil	40 ± 0.938
4	Cystatin + 0.64 *µ*M donepezil	38 ± 0.772
5	Cystatin + 0.96 *µ*M donepezil	24 ± 0.932
6	Cystatin + 1.6 *µ*M donepezil	15 ± 0.680

All data are expressed as mean ± S.E. for three different sets of experiments; statistical significance was conducted employing one way ANOVA. A probability level of 0.05 was selected showing that results are significant.

## Abbreviations

BC: Brain cystatin
AChE: Acetyl cholinesterase
ACh: Acetylcholine
AD: Alzheimer's disease.
